# Detection and characterization of drug-resistant conferring genes in *Mycobacterium tuberculosis* complex strains: A prospective study in two distant regions of Ghana

**DOI:** 10.1016/j.tube.2016.05.014

**Published:** 2016-07

**Authors:** I.D. Otchere, A. Asante-Poku, S. Osei-Wusu, A. Baddoo, E. Sarpong, A.H. Ganiyu, S.Y. Aboagye, A. Forson, F. Bonsu, A.I. Yahayah, K. Koram, S. Gagneux, D. Yeboah-Manu

**Affiliations:** a Noguchi Memorial Institute for Medical Research, University of Ghana, Accra, Ghana; b Department of Biochemistry, Cell and Molecular Biology, University of Ghana, Accra, Ghana; c Chest Clinic, Korle-BU Teaching Hospital, Accra, Ghana; d Ghana Health Service, Ministry of Health, Accra, Ghana; e Chest Department, Tamale Teaching Hospital, Tamale, Ghana; f Swiss TPH, Basel, Switzerland; g University of Basel, Basel, Switzerland

**Keywords:** Drug resistant, Spoligotype, Compensatory, Mutations, Virulence

## Abstract

We spoligotyped and screened 1490 clinical *Mycobacterium tuberculosis* complex strains from Northern and Greater Accra regions of Ghana against INH and RIF using the microplate alamar blue phenotypic assay. Specific drug resistance associated genetic elements of drug resistant strains were analyzed for mutations. A total of 111 (7.5%), 10 (0.7%) and 40 (2.6%) were mono-resistant to INH, RIF, and MDR, respectively. We found the Ghana spoligotype to be associated with drug resistance (INH: 22.1%; p = 0.0000, RIF: 6.2%; p = 0.0103, MDR: 4.6%; p = 0.0240) as compared to the Cameroon spoligotype (INH: 6.7%, RIF: 2.4%, MDR: 1.6%). The propensity for an isolate to harbour *katG* S315T mutation was higher in *M. tuberculosis* (75.8%) than *Mycobacterium africanum* (51.7%) (p = 0.0000) whereas the opposite was true for *inhApro* mutations; MAF (48.3%) compared to MTBSS (26.7%) (p = 0.0419). We identified possible novel compensatory INH resistance mutations in *inhA* (G204D) and *ahpCpro* (-88G/A and -142G/A) and a novel *ndh* mutation K32R. We detected two possible *rpoC* mutations (G332R and V483G), which occurred independently with *rpoB* S450L, respectively. The study provides the first evidence that associate the Ghana spoligotype with DR-TB and calls for further genome analyses for proper classification of this spoligotype and to explore for fitness implications and mechanisms underlying this observation.

## 1 Introduction

Tuberculosis (TB) remains an important global health problem, with close to 9 million new cases per year and a pool of approximately 2 billion latently infected individuals worldwide [1]. Of particular concern are the on-going epidemics of drug resistant TB (DR-TB), which threaten to make TB incurable. The main causative agents for human tuberculosis are *Mycobacterium tuberculosis* sensu stricto (MTBSS) and *Mycobacterium africanum* (MAF), both members of the *M. tuberculosis* complex (MTBC). Of particular interest to West Africa is MAF, which causes up to 50% of human TB in some countries [2].

Drug resistance to isoniazid (INH) and rifampicin (RIF), the two backbone-drugs of the directly observed treatment short-course (DOTS) regimen, can negatively affect the successful outcome of treatment of TB [3], [4]. The pro-drug INH requires activation by catalase peroxidase encoded by the *katG* of the target bacterium. Activated INH disturbs cell wall synthesis by binding to a 2-trans-enoyl-acyl carrier protein reductase (encoded by *inhA*) required for mycolic acid synthesis [5]. Rifampicin kills bacteria by halting the elongation of a nascent polynucleotide by occluding the path of the growing RNA through the polymerase [6]. *katG* (mostly S315T) and *inhApro* (mostly -15C/T) mutations are associated with high and low level INH resistance respectively [7]. Even though the *katG* mutation (inactive INH) and *inhApro* substitutions (overproduction of inhA) are the major causes of INH resistance, other loci such as *ndh, inhA* and *ahpCpro* have either been implicated with drug resistance or restoring the fitness cost associated with some high level resistance mutations [8], [9]. Some *rpoB* mutations alter the 3D structure of the *rpoB* hence affecting the conformation of the RIF-binding pocket which in effect prevents the rigid structure of RIF from binding to the bacterial polymerase to inhibit transcription [6], [10]. Even though *rpoB* mutations account for most RIF resistance in TB, mutations within the *rpoA* and *rpoC* have also been implicated with restoring the fitness cost associated with the acquisition of *rpoB* mutations [11]. Both *katG* S315T and *rpoB* S450L mutations (the most dominant INH and RIF resistance associated mutations respectively) have been associated with no/low fitness cost which may explain their dominance in clinical MTBC strains even though they confer high-level resistance [12], [13].

Though non-compliance of therapeutic regimen may contribute to the development of DR-TB, the contribution of the infecting bacteria cannot be underestimated. The Beijing strain of lineage 2 is associated with hyper-virulence and drug resistance even though reports on drug resistance are sometimes conflicting [14]. On the other hand, Lineage 1 is reported to be less virulent and negatively associated with high cost mutations, which are mostly responsible for high-level drug resistance [13], [14], [15]. This indicates that different genotypes of the MTBC may be associated with resistance to specific drugs and these associations may be driven by specific genetic alterations.

In this study, we screened MTBC strains isolated from pulmonary TB patients reporting to selected health centres from the Northern and Greater Accra Regions of Ghana for drug resistance to INH and RIF. We analysed specific genetic elements for DR associated mutations by DNA sequencing of implicated genes and looked for associations among drug resistance, bacterial genotypes and origin of the isolates.

## 2 Methodology

### 2.1 Ethical statement and participant enrolment

**Figure 1:**
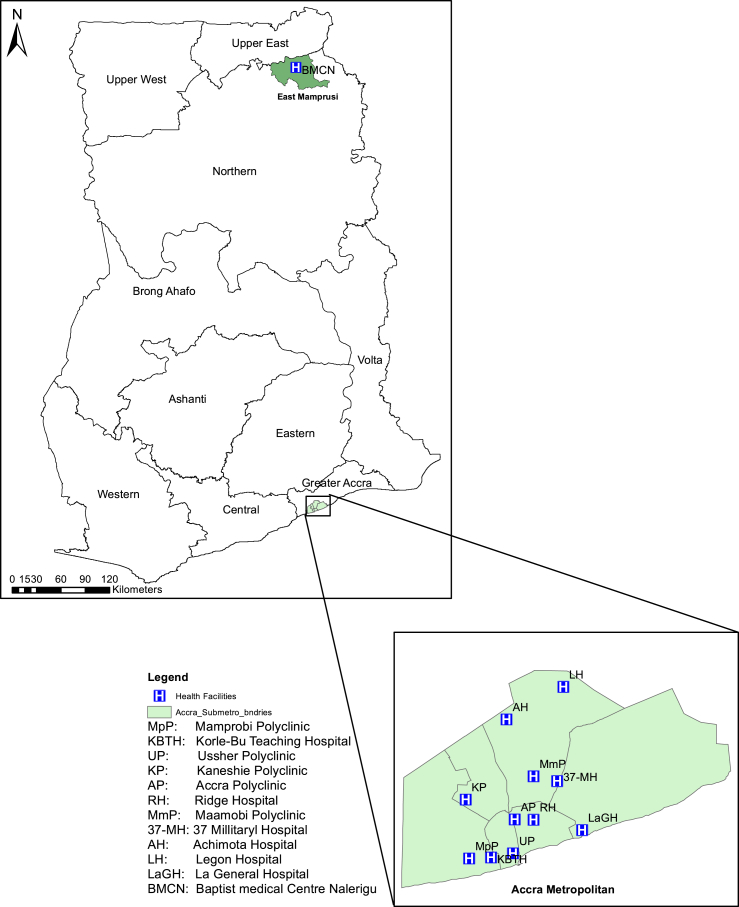
Map of Ghana showing the selected health centers from the two regions used for the study.

The Institutional Review Board of the Noguchi Memorial Institute for Medical Research (NMIMR) approved the study and its protocols. Following informed consent, consecutive sputum smear-positive TB cases who were not already taken anti-TB drugs or have been put on therapy for not more than 2 weeks were recruited from all TB diagnostic centres in the Accra Metropolitan (Southern Region) and the Mamprusi East (Northern Region) Health Administrations (Figure 1) from August 2012 to September, 2014. The standard procedure for sputum sample collection as outlined by the National Tuberculosis Control Program (NTP) for routine diagnosis of TB in Ghana was followed. Informed written consent was sought from all participants unless the participant was illiterate; in which case witnessed oral consent was used. Consent was sought from guardians of children below the age of 18 before enrolment into the study and in some cases child assent was also sought. Data collected from enrolled patients included age, sex, bacterial burden on day of diagnosis and TB treatment history.

### 2.2 Mycobacterial isolates

The sputum samples were decontaminated with 5% oxalic acid [16], and inoculated on Lowenstein-Jensen Media (supplemented with either glycerol or pyruvate) slants, incubated at 37 °C until growth was observed. Colonies from positive cultures were sub-cultured on similar media and incubated as above until confluent growth was observed. The isolates (1490) were confirmed MTBC by PCR amplification of IS*6110*, genotyped as MTBSS, MAF and/or *Mycobacterium bovis* by large sequence polymorphism (LSPs) detecting region of difference (RD) 4, 9 and 12 [17] and spoligotyping as described by Kamerbeek et al. [18].

### 2.3 Anti-TB drug susceptibility testing

Micro-plate alamar blue assay (MABA) for drug susceptibility testing (DST) was performed in clear-bottomed, 96-well micro-plates (Nunc International, Rochester, NY, US). Drugs stocks were prepared by following standard protocols [19], and MABA-DST set up by following a modification of the method described by Franzblau et al. [20].

In summary, mycobacterial inoculum was prepared by emulsifying a loop full of logarithmic growing mycobacteria in sterile Middlebrook 7H9 [Difco, Detroit, Michigan] broth supplemented with 0.2% glycerol [Sigma-Aldrich, Steinheim, Germany] and 0.001% casitone [Difco] (7H9-GC) and adjusted spectrophotometrically to OD_600_ = 1.0 containing approximately 1 × 10^8^ cfu/mL. This suspension was diluted 1:25 with 7H9-GC for inoculation. All perimeter wells of the plate were filled with 200 uL of sterile water to prevent evaporation of the content of inner wells. The test wells consisted of 7H9-GC/Tween 80 [Sigma-Aldrich] medium and respective concentration of testing drugs, (which were serially diluted to final concentrations of 0.03125–1.0 ug/mL and 0.0625 to 2.0 ug/mL for INH and RIF respectively) before inoculation with 100 μL of the mycobacterial suspension containing approximately 4 × 10^6^ cfu/mL. The plates were incubated at 37 °C for 7 days, and bacterial viability was tested with freshly prepared 1:1 v/v of alamar blue reagent [Trek Diagnostic Systems, OH, USA] and 10% Tween 80. The minimum inhibitory concentration (MIC) defined as the concentration of drug in the first blue well for each drug, was then recorded (S1). The critical drug concentration was ≤0.25 ug/mL for both drugs [20]. The positive and negative controls for each set were drug-free media inoculated with bacterial suspension and un-inoculated containing media only respectively.

### 2.4 Isolation of genomic DNA

The protocol used for the extraction of genomic DNA was a synthesis of the protocols of Van Soolingen et al. [21] and Käser et al. [22]. Briefly, the mycobacterial cell wall was disrupted by adding lysozyme (50 μL lysozyme of 10 mg/mL) vortexed and incubated overnight, followed by addition of 75 μL of 10% SDS, 10 μL proteinase K (20 mg/mL), vortexed softly and incubated 15 min at 65 °C. After, the incubation, 100 μL of 5 M NaCl was added, followed by 100 μL CTAB which was pre-warmed at 65 °C. After vortexing, the extracted DNA was purified by chloroform/isoamyl alcohol extraction. The DNA contained in the upper phase was precipitated with isopropanol and washed with ethanol. The dried DNA was then re-suspended in 100 uL Tris EDTA buffer and stored at 4 °C until use.

### 2.5 PCR amplification and DNA sequencing of genetic elements

Drug resistant isolates were used for targeted sequencing analyses. Eight drug resistance associated genetic elements (*rpoB*, *rpoA* and *rpoC* for RIF resistant and *inhApro*, *ahpCpro, inhA, ndh* and *katG* for INH resistant strains) were analysed for mutations.

All PCR reactions were set up as previously described [23]. In summary, each set up contained 3 μL of 10X buffer, 1.8 μL of 15 mM MgCl_2_, 3 μL of Q solution, 0.6 μL of 10 mM dNTP mix, 1.8 μL of each primer, 0.2 μL of Hot-start Taq polymerase from Qiagen, 14.8 μL of nuclease-free water and 3 μL of template DNA. Cycling conditions were: initial denaturation at 95 °C for 15 min and 35 cycles of denaturation at 96 °C for 1 min, annealing at specific Tm (Table 1) for 1 min, extension at 68 °C for 1 min and final extension at 72 °C for 10 min. The obtained amplicons were shotgun sequenced in both directions by outsourcing to Macrogen Europe.

### 2.6 Data analyses

The DNA sequences were screened for possible mutations by comparing with the standard strain; H37Rv genome downloaded from the tuberculist database [24] using the Staden software [25]. DNA sequencing was repeated for all isolates with novel mutation(s) for verification. We compared the proportion and kind of mutations in MTBSS and MAF as well as the geographic origin of the isolates. The level of resistance between the different genotypes and the kind of mutation were compared with fishers exact tests using stata statistical package [26], with significance threshold set to *P* < 0.05.

## 3 Results

### 3.1 Clinical data of drug-resistant patients

Among the 161/1490 (10.8%) patients from whom MTBC strains resistant to at least one drug were isolated, 114 (70.8%) and 47 (29.2%) were males and females respectively. Drug-resistant TB patients up to 50 years were 113 (70.2%) whereas those above 50 years were 48 (29.8%). Whereas 128 (79.5%) of the DR-TB patients were having TB for the first time, 33 (20.5%) had been treated for TB before the current episode. Bacterial burden of the DR-TB patients stood at 9 (5.6%) scanty, 83 (51.6%) 1+, 29 (18.0%) 2+ and 40 (24.8%) 3+. 12.8% of drug resistant TB patients as compared to 12.2% of drug susceptible TB patients were HIV positive. Whereas 140 (87.0%) of the DR-TB patients were from the South representing 10.4% of TB patients reporting to the selected hospitals in the region, 21 (13.0%) were from the North representing 15.0% of the patients reporting to the selected hospital from that region.

### 3.2 Drug-susceptibility rates

One thousand four hundred and ninety isolates (MTBSS: 1208; 81.1%, MAF: 272; 18.3% and *M. bovis*: 10; 0.7%) were tested and of these, 111 (7.5%), 10 (0.7%), 40 (2.6%) and 161 (10.8%) were INH mono-resistant (INH^r^), RIF mono-resistant (RIF^r^), multi-drug resistant (MDR) and resistant to at least one drug (ANY^r^) respectively. Comparing these results to a previous report from Ghana [27], indicates that the level of drug resistance has remained fairly low over the past 8 years (OR = 1.11, 95% CI = 0.79–1.52, p = 0.5186). There was no statistical association of specific DR-TB with the two regions understudied (Table 2). We found 90/1208 (7.5%), 19/272 (7.0%) and 2/10 (20%) of the MTBSS, MAF and *M. bovis* respectively were INH^r^. None of the MAF isolates were RIF^r^, however, there were 9 (0.7%) MTBSS and 1 (10.0%) *M. bovis* resistant to only RIF. On the other hand, the MDR strains were 10 (3.7%) MAF and 30 (2.5%) MTBSS. There was no difference between the DR rates of MAF and MTBSS (Table 3). When the strains were further stratified into spoligotypes, the DR rates as summarized in Table 4 indicate a high proportion of DR among the Ghana spoligotype of Lineage 4. For example, there were 7.4% of Cameroon spoligotype as compared to 23.6% of the Ghana spoligotype with resistance to at least one drug (OR = 0.26; CI 0.17–0.41; p = 0.0000).

### 3.3 Drug resistance conferring mutations

Mutations were found in all targets analysed but *rpoA* of RIF resistant isolates (Table 5) with the *katG* S315T (70.9%) and *rpoB* S450L (52.0%) as the dominant INH and RIF resistance-associated mutations respectively followed by *inhApro* -15C/T (15.9%) and *rpoB* D435V (22.0%). As shown (Table 6), 91 (75.8%) of INH resistant MTBSS compared to 15 (51.7%) MAF harboured *katG* mutations (OR: 3.73, 95% CI: 1.56–9.09, p = 0.0013). In contrast, within the *inhApro*, 32 (26.7%) MTBSS as compared to 14 (48.3%) INH resistant MAF harboured mutations (OR: 0.39, 95% CI: 0.16–0.98, p = 0.0419). Analysis of other targets associated with INH resistance, found the proportion of INH resistant MAF with *ndh* (5; 17.2%) mutations to be higher than MTBSS (1; 0.8%) (OR: 0.04, 95% CI: 0.00–0.39, p = 0.0011). Mutations within the *inh*A locus, and *ahpCpr*o regions were, 17 (14.2%) and 2 (1.7%) in MTBSS as compared to 1 (3.5%) and 1 (3.5%) MAF (p = 0.1999 and p = 0.4802 respectively), All the 16 isolates with the novel i*nhA* mutation (G204D) were MTBSS Lineage 4 spoligotypes (11 Ghana, 3 Cameroon, 1 Uganda I and 1 LAM). MTBSS strains with *rpoB* mutations were 38 (97.4%) as compared to 8 (80.0%) MAF (p = 0.1018) as shown in Table 6. The only RIF resistant bovine strain had two *rpoB* mutations Q432P and I491S (Table 5; S1).

Exploring the mutations in the genetic elements under study for association among the Lineage 4 spoligotypes (Table 7) found 25.6% of the Ghana spoligotype to harbour *inhA* mutations as compared to 7.4% of the Cameroon spoligotype (OR = 4.23, 95% CI = 1.13–19.82, p = 0.0220). In addition, all the 3 strains with the novel *ndh* mutation (V117I) belonged to MAF West Africa 2. The 2 strains with *rpo*C G332R were Cameroon spoligotypes whereas the 3 strains with *rpo*C V483G were Ghana spoligotype but were all carrying *rpoB* S450L.

## 4 Discussion

The objectives of this study were 1) to determine the drug (INH and RIF) susceptibility profile of MTBC strains, 2) to probe the genomes of DR isolates for mutations and 3) to compare the identified mutations between species. The main findings from this prospective study show that 1) the rate of INH^r,^ RIF^r^ and MDR TB has remained fairly constant over the years with relatively high proportion of INH^r^ as compared to RIF^r^, 2) the Ghana spoligotype of Lineage 4 is associated with drug resistance 3) that INH resistant MTBSS are more likely to harbour *katG* mutations as compared to INH resistant MAF which preferred i*nhApro* mutations, and 4) that RIF resistant Cameroon and Ghana spoligotypes of Lineage 4 may harbour different compensatory *rpoC* mutations even though they share the same drug resistant *rpoB* mutation.

The proportion of INH^r^, RIF^r^ and MDR among the close to 1500 isolates analysed were 7.5%, 0.7% and 2.6% respectively which was found not to be significantly different from two previous reports [23], [27]. This indicates that the rate of MDR and RIF mono resistant TB in the country has remained fairly constant and low over the years indicating some success for the National control programme. However, the 111 (7.5%) INH mono-resistance as compared to 10 (0.7%) RIF mono-resistance suggests that emergence of INH resistance predates RIF resistance towards generation of MDR-TB. This finding has important implications if GeneXpert is used as a surrogate for detection of MDR-TB as most INH-mono-resistant cases would be treated as susceptible with first-line regimen that contains INH and could render the first-line regimen ineffective.

Stratifying the isolates by geographical region shows that the northern part of the country has higher proportion of MDR-TB compared to the south and the national average of approximately 2% according to the national tuberculosis control program (personal communication with the director). This result calls for intensifying control activities in Northern Ghana and probably other rural communities as most of the Global fund activities are centred in the urban areas especially the Accra Metro Health Directorate. However, consistent with an earlier study [23], there was no difference between the proportion of MTBSS and MAF that were drug resistant (Table 2). This result however, seems to contradict a study in Ghana that found MTBSS to be more associated with drug resistance as compared to MAF [28]. However, resistance to streptomycin (which was not included in the current study) drove the association found in that study. The Ghana spoligotype of MTBSS Lineage 4, which is the second most dominant Lineage 4 spoligotype in Ghana [23], [28], was found to be associated with drug resistance whether compared to the most dominant Cameroon spoligotype or the two lineages of MAF (Table 3). This is the first report that associates the Ghana spoligotype with drug resistance in any of the West African countries where this genotype is prevalent. This association is similar to the association of the Beijing genotype of Lineage 2 in some geographical settings [28], [29], [30], [31], [32]. However, whereas the Beijing genotype is defined by other genotyping tools in addition to spoligotyping, the Ghana spoligotype is merely defined by spoligotyping, which calls for further genomics studies for proper classification. Drug resistance mutations in most bacteria, including MTBC is often associated with a fitness cost, and therefore the ability of a strain to develop and transmit effectively is influenced by the genetic background that mitigate against the cost. For instance, the Beijing genotype has been associated with increased virulence and compensatory mutations [32], [33], [34], [35], [36]. Drawing from the Beijing experience, it may be appropriate to infer that the Ghana spoligotype has developed the capacity to develop drug resistance through some as-yet-unidentified evolutionary compensatory mechanism. As high rate of drug resistance is somehow linked with virulence [32], [33], [34], [35], detailed comparative genomics and phenotypic investigations involving the Ghana spoligotype would highlight the probable evolutionary forces driving this association with drug resistance. This finding partly explains, the higher level of DR-TB observed in the Northern region of Ghana probably confounded by the dominance of the Ghana spoligotype which is found in higher proportions in that part of the country as compared to the South [Yeboah-Manu et al., manuscript submitted]. This observation could also be due to a local transmission of resistant Ghana spoligotype strains instead of emergence of resistance among patients under therapy similar to an earlier report by Eldholm et al. [37]. This however, would require whole genome sequencing of the drug resistant strains for clarity.

The finding that INH resistant MTBSS clinical strains have higher preference for *katG* mutations as compared to INH resistant MAF strains which preferred *inhApro* mutations compares with an earlier report of a similar study carried out in Ghana [27], and suggests possible preference for lineage-specific drug-resistance mutations. However, whereas that study hypothesized that *inhApro* mutation -102G/A which has not been associated with drug resistance was likely to be MAF species-specific SNP, the mutation was later detected in some INH resistant MTBSS strains harbouring either *katG* S315T or *inhApro* -15C/T and hence challenged the MAF species-specificity of this SNP [23]. In this current study, the mutation was again found in both species (S1), thus confirming the earlier hypothesis that the mutation could not be species-specific. We however hypothesize that the -102G/A, together with -47G/C and -8T/C SNPs which were also detected in the previous studies are more likely to be compensatory mutations instead of specific phylogenetic markers. This hypothesis is supported by the fact that the 3 SNPs mentioned above were found concurrently with either *katG* S315T or *inhApro* -15C/T in INH resistant strains or alone in INH susceptible strains and were not species-specific (S1). Nevertheless, this hypothesis requires further work for confirmation or otherwise. However, if this hypothesis is proven to be true, it would have negative impact on the WHO approved MTBDR*plus* line probe assay which has the *inhApro* -8T/C SNP as one of the markers for INH resistance. We also report of a less prevalent *inhApro* mutation -17G/C which was earlier reported in some Ghanaian isolates [27], however unlike the earlier report, this study found the affected isolate had no other mutation in all the other targets analysed suggesting that it could be responsible for the observed resistance.

In addition, there was a novel *inh*A mutation (G204D) which may be a compensatory mutation for the acquisition of *katG* S315T mutations in MTBSS whereas another novel mutation in *ndh* (K32R) could be a drug resistance mutation (Table 4). These conclusions were drawn from the observation that all the 16 INH resistant isolates with the *inhA* G204D mutation also had the *katG* S315T mutation whereas the only isolate with the *ndh* K32R mutation had no other mutation in all the targets analysed apart from another novel mutation V117I in the same gene. The additional *ndh* mutation V117I may be a compensatory mutation because it was also detected in four additional INH resistant strains with *katG* S315T. However, whereas all the 3 isolates with the *ndh* V117I mutation were MAF Lineage 6, the *inhA* G204D was detected in 16 MTBSS Lineage 4 strains of four different spoligotypes with *katG* S315T hence ruling out the possibility of a phylogenetic marker (S1). Even though this novel mutation was found in four genotypes of Lineage 4, it was found to be more associated with the Ghana spoligotype as compared to the Cameroon (OR = 0.24, 95% CI = 0.05–0.88, p = 0.0220). If this mutation is verified as compensatory, the association with the Ghana spoligotype may explain the positive selection and fixation of INH resistance mutations in the Ghana spoligotype, similar to the situation with the Beijing genotype [32].

The differences in *rpoC* mutation between RIF resistant Cameroon and Ghana spoligotypes of Lineage 4 is in line with earlier work [37], which suggested that under similar physiological conditions, the genetic background of a particular strain may select for specific mutations. However, the physical environment of the strains may be the driving force for selection and fixation of these mutations irrespective of the genetic background [38]. Interestingly, these reports are all based on *in vitr*o generated mutant strains suggesting that, under clinical conditions, the two proposals may apply. Even though the *rpoC* mutations G332R and V483G were found only in Ghana and Cameroon spoligotypes respectively, they were not spoligotype-specific (S1), thus ruling out the possibility of them being phylogenetic markers. In addition, the V483G mutation has been shown to occur at the interface between the *rpoB*, *rpoA* and *rpoC* of the bacterial polymerase, hence predicted with high confidence to be a compensatory mutation [11]. This current work also reaffirms the dominance of the *rpo*B S450L mutations among clinical RIF resistant strains and emphasizes the possible role played by compensatory mutations for the fixation of this high level RIF resistance associated mutation among clinical isolates [11].

The major limitation of this paper rests on the classification of the Ghana genotype of Lineage 4 by spoligotyping alone. However, the slow generation time and no/low recombination of *M. tuberculosis* complex strains suggest this observation may even hold with whole genome sequence data. In addition, the number of isolates from the north was relatively small. Furthermore, all the genomic regions analyzed were within the previously described hotspots [39] of the distinct targets analyzed and did not probe new regions. Nevertheless, the information reported here is useful for efficient management of TB in Ghana in that it provides confidence in newly diagnostic tools such as GeneXpert to support detection of RIF resistance. It also calls for more intensive control activities in the rural areas as we found more resistant cases in the rural district in northern Ghana compared to the urban Accra Metro. The findings also reaffirm the importance of understanding the genomic diversity of the MTBC in the fight against drug resistant TB.

## 5 Conclusion

This study reports an association between specific MTBC spoligotype and drug resistance in a geographical certain with most of the MTBC species circulating in appreciable proportions. The study provides the first evidence that implicate the Ghana spoligotype as the driver of DR-TB in Ghana and calls for further genome analyses to best characterize this spoligotype, explore for fitness and mechanisms underlying this observation. This work also reports that even though two different genotypes may have the same drug resistant mutation, they may harbour different compensatory mutations suggesting sub-lineage specific epistatic interaction between drug resistance conferring and non-drug resistant conferring mutations. The study finally confirms an earlier report that associated *M. tuberculosis* sensu stricto with *katG* and *M. africanum* with *inhApro* for high-level and low level INH resistance respectively.

## Figures and Tables

**Table 1:** Primers and conditions for PCR amplification of genetic element.

No.	Name	Sequence (5′–3′)	Tm (°C)	Target	Product size
1	rpoA1F	GCATTCCAGTCGATTCCATC	57	*rpoA*	560 bp
2	rpoA1R	CCAAGATCGCCTTCTGATGT
3	rpoA2F	GGACGTCGAAAGGAAGAAGA	57	*rpoA*	542 bp
4	rpoA2R	GTCTCCACGTCCAGGATCAG
5	rpoBF	GTAGTCCACGCCGTAAACGG	65	*rpoB*	601 bp
6	rpoBR	ACGTCCATGTAGTCCACCTCAG
7	rpoCF	CGAAAACCTCTACCGCGAAC	64	*rpoC*	650 bp
8	rpoCR	GCGACAGGATGTTGTTGGAG
9	inhAproF	ATCACCACCGCCGCTGAAGC	65	*inhApro*	650 bp
12	inhAproR	GTTCGGGTACCCGGGAATG
11	inhA1F	CTACATCGACACCGATATGAC	57	*inhA*	600 bp
12	inhA1R	GACCGTCATCCAGTTGTAG
13	inhA2F	GCATCAACCCGTTCTTCGAC	57	*inhA*	550 bp
14	inhA2R	TAATGCCATTGATCGGTGATAC
15	ahpCproF	ACCACTGCTTTGCCGCCACC	70	*ahpCpro*	340 bp
16	ahpCproR	CCGATGAGAGCGGTGAGCTG
17	katGF	CCAGCGGCCCAAGGTATC	65	*katG*	820 bp
18	katGR	GCTGTGGCCGGTCAAGAAGAAGT
19	ndhF	ATCACCACCGCCGCTGAAGC	64	*ndh*	589 bp
20	ndhR	GTTCGGGTACCCGGGAATG

**Table 2:** Distribution of drug resistance among 1490 MTBC isolates from two distant regions of Ghana.

Resistance	Total (1490)	Accra Metro (1350)	Mamprusi East (140)	p-value
INH^r^	111; 7.45%	99; 7.33%	12; 8.60%	0.6108
RIF^r^	10; 0.67%	8; 0.59%	2; 1.40%	0.2404
MDR	40; 2.64%	33; 2.44%	7; 5.00%	0.0924
ANY	161; 10.80%	140; 10.37%	21; 15.00%	0.0850

**Table 3:** Distribution of drug resistance by the human adapted MTBC.

Drug	Total (1480)	MTBSS (1208)	MAF (272)	p-value	95% CI
INH^r^	109; 7.4%	90; 7.5%	19; 6.9%	0.8979	0.63–1.89
RIF^r^	9; 0.6%	9; 0.7%	0; 0.0%	0.3794	0.44–Inf
MDR	40; 2.7%	30; 2.5%	10; 3.7%	0.2988	0.31–1.55
ANY^r^	158; 10.7%	129; 10.7%	29; 10.7%	0.9999	0.65–1.59

**Table 4:** Comparing the proportion of drug resistant Ghana spoligotype of MTBSS to other prominent spoligotypes in the Ghana.

Resistance	Cameroon (806)	Ghana (195)	OR	95% CI	p-value
INH	54 (6.7%)	43 (22.1%)∗	0.25	0.16–0.40	0.0000
RIF	19 (2.4%)	12 (6.2%)∗	0.37	0.17–0.85	0.0103
MDR	13 (1.6%)	9 (4.6%)∗	0.34	0.13–0.91	0.0240
ANY	60 (7.4%)	46 (23.6%)∗	0.26	0.17–0.41	0.0000
	MAF WA 1 (165)	Ghana (195)			
INH	25 (15.2%)	43 (22.1%)	0.63	0.35–1.12	0.1059
RIF	10 (6.1%)	12 (6.2%)	0.98	0.37–2.56	0.9998
MDR	10 (6.1%)	9 (4.6%)	1.33	0.47–3.81	0.6384
ANY	25 (15.2%)	46 (23.6%)∗	0.58	0.32–1.02	0.0472
	MAF WA 2 (107)	Ghana (195)			
INH	6 (5.6%)	43 (22.1%)∗	0.21	0.07–0.52	0.0001
RIF	1 (0.9%)	12 (6.2%)∗	0.144	0.00–1.00	0.0372
MDR	1 (0.9%)	9 (4.6%)	0.19	0.00–1.45	0.1040
ANY	6 (5.6%)	46 (23.6%)∗	0.19	0.06–0.48	0.0000

MAF WA 1: *M. africanum* West Africa 1 (Lineage 5).

MAF WA 2: *M. africanum* West Africa 2 (Lineage 6).

∗ Significantly higher.

**Table 5:** Identified mutations from drug resistant MTBC.

Gene (No. of isolates)	DNA mutation	Isolates with specific SNP	Amino acid change	Other mutations
*inhApro* (151)	-8T/C	5 (3.3%)	–	5: *katG* S315T, 2: *ndh* V117I
−15C/T	24 (15.9%)		8: inhApro -102G/A
−17G/C	1 (0.7%)	–	
−102G/A	16 (10.6%)	–	8: *katG* S315T, 8: *inhApro* -15C/T
−47G/C	1 (0.7%)		*katG* S315T
*katG* (151)	G944C	107 (70.9%)	S315T	16: *inhA* G204D, 8: *inhApro* -102G/A: 5: *inhApro* -8T/C, 1: *inhApro* -47G/C, 2: *ndh* V117I, 2: *ahpCpro* -88G/A, 1: *ahpCpro* -142 G/A
*inhA* (151)	G611A∗	16 (10.6%)	G204D∗	16: *katG* S315T
T233C	1 (0.7%)	V78A	*ndh*: K32R; *aphCpro*: -54C/T
*ahpCpro* (151)	−54C/T	1 (0.7%)	–	*ndh*: K32R; *inhA:* V78A
−88G/A∗	2 (1.3%)	–	2: *katG;* S315T
−142G/A∗	1 (0.7%)	–	*katG*: S315T
*ndh* (151)	G349A	4 (2.6%)	V117I∗	*inhApro;* -8T/C *katG;* S315T
C411T	1 (0.7%)	G137G	*katG*; S315T
A95G & G349A	1 (0.7%)	K32R∗ & V117I∗	*ahpCpro*: -54C/T, *inhA:* V78A
*rpoB* (50)	A1295C	1 (2.0%)	Q432P	
A1295C & T1472G	1 (2.0%)	Q432P & I491S†	
C1294A	1 (2.0%)	Q432K	
A1304T	11 (22.0%)	D435V	
G1303T	1 (2.0%)	D435Y	
C1332T	1 (2.0%)	S441L	
A1334G	2 (4.0%)	H445R	
CA1333/4TG	2 (4.0%)	H445C	
C1333G	2 (4.0%)	H445D	
C1333T	1 (2.0%)	H445Y	
C1349T	26 (52.0%)	S450L	2: *rpoC* G332R, 3: *rpoC* V483G
*rpoC* (50)	G994C	2 (4.0%)	G332R	*rpoB*; S450L
T1448G	3 (6.0%)	V483G	*rpoB*; S450L

The 50 RIF resistant strains include the 49 RIF mono-resistant and/or MDR MTBSS and MAF strains in addition to the single RIF mono-resistant *Mbovis*. Similarly, the 151 INH Resistant strains include the 109 INH mono-resistant and 40 MDR MTBSS and MAF in 2 INH mono-resistant *M.bovis*.

∗ Novel Mutation.

† Previously described in RIF resistant *E. coli*.

**Table 6:** Distribution of mutations by human adapted MTBC species.

Target	MTBSS	MAF	OR	95% CI	p-value
*katG*	91; 75.8%∗	15; 51.7%	3.73	1.56–9.09	0.0013
*inhApro*	32; 26.7%	14; 48.3%∗	0.39	0.16–0.98	0.0419
*inhA*	17; 14.2%	1; 3.5%	4.59	0.66–199.77	0.1999
*ahpCpro*	2; 1.7%	1; 3.5%	0.48	0.02–28.99	0.4802
*ndh*	1; 0.8%	5; 17.2%∗	0.04	0.00–0.39	0.0011
*rpoB*	38; 97.4%	8; 80.0%	8.88	0.42–572.05	0.1018
*rpoC*	5; 12.8%	0; 0.0%	Inf	0.22-Inf	0.5834

∗ Significantly higher.

**Table 7:** Comparing mutation sites among Lineage 4 Spoligotypes.

	Cameroon (54)	Ghana (43)	OR	95% CI	p-value
*katG*	39; 72.2%	32; 74.4%	0.89	0.32–2.42	0.9998
*inhApro*	14; 25.9%	9; 20.9%	1.32	0.46–3.92	0.6357
*inhA*	4; 7.4%	11; 25.6%∗	0.24	0.05–0.88	0.0220

∗ Significantly higher.
